# Development of new immunotherapy treatments in different cancer types

**Published:** 2016

**Authors:** DL Stanculeanu, Zob Daniela, A Lazescu, R Bunghez, R Anghel

**Affiliations:** * “Prof. Dr. Al. Trestioreanu” Institute of Oncology, Bucharest, Romania; ** Clinical Education Department, Oncology Discipline, “Carol Davila” University of Medicine and Pharmacy, Bucharest, Romania

**Keywords:** immunotherapy, cancer, arthrodesis, new treatments, clinical studies

## Abstract

Cancer immunotherapy involves the use of therapeutic modalities that determine a manipulation of the immune system by using immune agents such as cytokines, vaccines, cell therapies and humoral, transfection agents. Immunotherapy of cancer has to stimulate the host’s anti-tumor response by increasing the effector cell number and the production of soluble mediators and decrease the host’s suppressor mechanisms by inducing tumor killing environment and by modulating immune checkpoints. Immunotherapy seems to work better in more immunogenic tumors. Making a review of literature, the article presents the new immunologic treatments in cancers less presented in the latest conferences, cancers in which, immunotherapy is still under investigation. Bladder cancer was the first indication for which immunotherapy was used in 1970. A promising clinical research in bladder cancer is the use of immune checkpoint inhibitors. Although breast cancer is considered immunologically silent, several preclinical and clinical studies suggested that immunotherapy has the potential to improve the clinical outcomes for patients with breast cancer. Cervical cancer, brain cancer, head and neck cancer and colorectal and esophageal cancers are cancer types for which new immune-based cancer treatments are currently under development. Recent agents used in clinical trials will be described in before mentioned cancers.

Immunotherapy in cancer is a type of treatment discovered in the 1970s, with the onset of bladder cancer therapy with BCG [**1**] and IFN therapy in malignant melanoma. Various immune therapies such as IL 2 cytokine used in solid tumors like melanoma were discovered. A period of decline of these therapies followed, with powerful side effects and minor results. Along with studying the mechanisms of the immune response, there are cells involved in the immune response, mediators that cause stimulation or inhibition of the immune response, developing new therapies.

Cancer immunotherapy involves the use of therapeutic modalities that lead to a manipulation of the immune system by using immune agents such as cytokines, vaccines, cell therapies, and transfection agents (**Fig. 1**).

Immunotherapy of cancer: stimulates the host’s anti-tumor response by increasing the effector cell number (like DC based vaccines) and production of soluble mediators (like increased tumor cell immunogenicity) and decreases the host’s suppressor mechanisms by inducing tumor killing environment and by modulating immune checkpoints [**2**] (**Fig. 2**). Cancer immunotherapy presupposes treatments that enhance the innate powers of the immune system to fight cancer and represents the most promising new cancer treatment approach since the development of the first chemotherapies in the late 1940s.

**Fig. 1:**
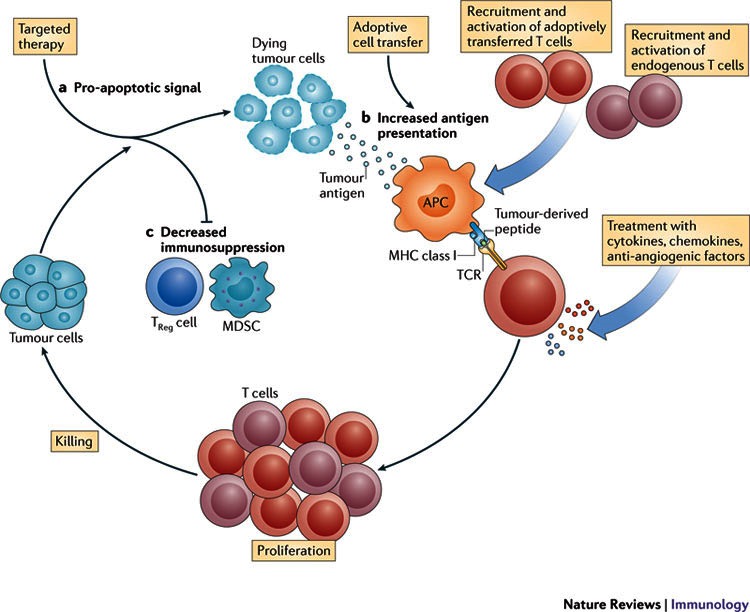
Therapeutic modalities that lead to a manipulation of the immune system by using immune agents such as cytokines, vaccines, cell therapies, and transfection agents

Immunotherapy seems to work better in more immunogenic tumors. The article presents some new immunologic treatments in different types of cancers less presented in the latest conferences, cancers in which, immunotherapy is still under investigation.

Bladder cancer was the first indication for which an immunotherapy was used in 1970. Currently, there are a number of additional immune-based bladder cancer treatments under development. Most bladder cancers start in the transitional epithelial cells and are represented by urothelial carcinoma [**3**].

The overall 5-year survival rate for bladder cancer is 77%, with differences according to stage, and this rate has not changed significantly over the last years, a period during which no new drugs for bladder cancer were discovered.

**Fig. 2:**
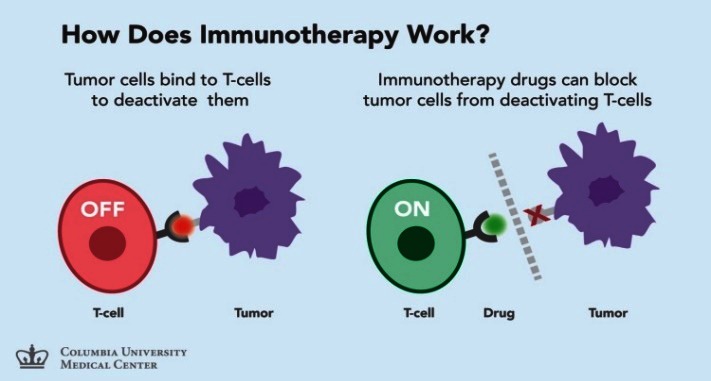
Immunotherapy of cancer

Treatment for non-muscle invasive bladder cancer is represented by the surgical removal of the tumor followed by intravesical chemotherapy, usually Epirubicin being administered at 8 hours after surgery. Patients with a lower risk of disease progression may undergo surveillance or additional intravesical chemotherapy. Patients with moderate- to high-grade disease often receive intravesical immunotherapy with bacillus Calmette-Guérin (BCG). The standard treatment according to the guidelines for patients with muscle invasive bladder cancer includes cisplatin-based chemotherapy regimens, neoadjuvant administration followed by surgical removal of the bladder or radiation therapy and concomitant chemotherapy. Recurrent and metastatic bladder cancer is treated with chemotherapy regimens that include methotrexate, vinblastine, doxorubicin, and cisplatin (MVAC) or gemcitabine plus cisplatin (GC), two regimens with the same response rates. 

A promising clinical research in bladder cancer is the use of immune checkpoint inhibitors (**Fig. 3**) which work by targeting molecules that serve as checks in the regulation of immune responses and block inhibitory molecules or activate stimulatory molecules and enhance pre-existing anti-cancer immune responses. There are studies using Nivolumab, Ipilimumab, and Pertuzumab in metastatic bladder cancer that are still recruiting [**4**].

**Fig. 3:**
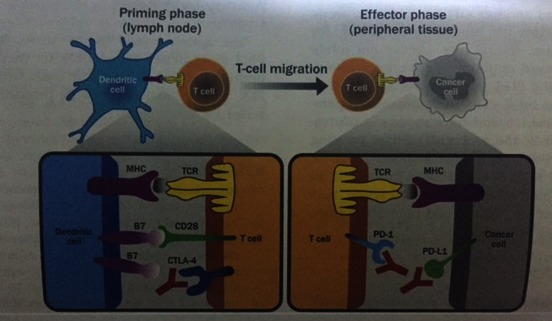
From “Understanding the Rationale for Immunotherapy in Non-Small Cell Lung Cancer”- Tumor immunotherapy: role of immune checkpoint inhibitors

Therapeutic Vaccines (**Fig. 4**) elicit an immune response against tumor-specific or tumor-associated antigens. Several trials of vaccines are currently enrolling patients: a therapeutic vaccine made from a human bladder cancer cell line that has been irradiated and engineered to express soluble gp96, a chaperone protein being used in phase II of the trial that is currently enrolling patients with high-risk, non-muscle invasive bladder cancer who have completed surgery. A phase I study is testing a fusion protein vaccine with or without the biological therapy sirolimus in patients with a variety of solid tumors, including recurrent and metastatic bladder cancer [**5**].

**Fig. 4:**
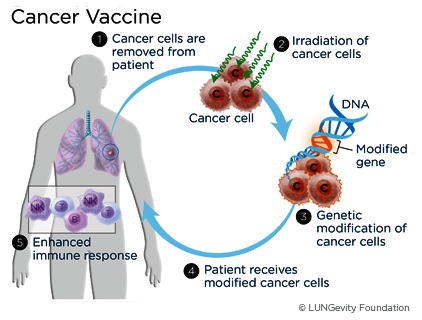
Therapeutic cancer vaccines

Cytokines are messenger molecules that are able to help controlling the growth and activity of immune system cells. Monoclonal antibodies are molecules generated in the laboratory that can target specific antigens on tumors. Combining them seems to be a good immunologic treatment. There is a fusion of the cytokine interleukin-2 (IL-2) and an antibody that recognizes peptides on the surface of the tumor cells that was studied in clinical trials. Treatment with IL-2 can enhance the activity of the immune system against tumors and, by linking IL-2 to the antibody, ALT-801 can target IL-2 to cancer cells [**6**].

Oncolytic virus therapy uses a modified virus that can make tumor cells self-destruct and release antigens therefore generating a greater immune response against the cancer. The best-known oncolytic virus is an oncolytic adenovirus that also expresses the immune stimulating cytokine GM-CSF. This oncolytic adenovirus is administered intravesical and further enhances the anti-tumor immune response, being tested in a phase II/ III study in patients with carcinoma in situ (CIS) of the bladder or with non-muscle invasive bladder cancer plus CIS of the bladder and who have failed BCG therapy [**7**-**9**].

Breast cancer is one of the major cancer types that affect the economy of all countries. Some new treatments have been developed and approved in the last years. New immune-based cancer treatments are currently under development [**10**]. Breast cancer is the most commonly diagnosed cancer among women (**Fig. 5**), 12 percent of all cancers diagnosed globally being represented by breast cancer each year. Breast cancer is the second leading cause of cancer-related death among women. The overall 5-year survival rate for breast cancer is of 90% - with differences according to stage.

**Fig. 5:**
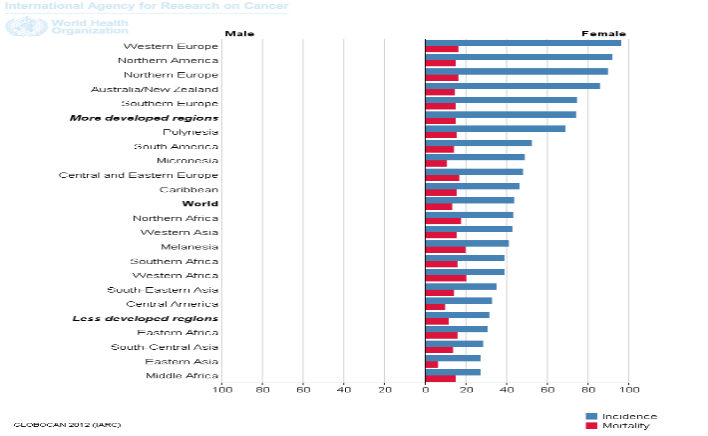
Breast cancer incidence and mortality rates in males and females

The increased risk for breast cancer is associated, among others, with a personal or family history of the disease and inherited gene mutations in BRCA1 and BRCA2 breast cancer susceptibility genes. Approximately 5%-10% of all breast cancer cases have an inherited predisposition to develop breast cancer.

Breast cancer treatment involves surgery, which, may be followed by chemotherapy, radiation therapy, or targeted therapy (including hormone therapy). Cancers over-expressing HER2 (HER2 3+, or FISH-positive) may be treated with trastuzumab and pertuzumab and, in the case of advanced cancer, lapatinib or trastuzumab emtansine. Of these, the newest treatment options are pertuzumab and trastuzumab emtansine. For patients with ER+ or PR+ breast cancer refractory to endocrine therapy, or patients who have triple negative breast cancer, targeted therapeutic options remain quite limited.

Breast cancer is considered immunologically silent, but several preclinical and clinical studies suggested that immunotherapy has the potential to improve the clinical outcomes for patients with breast cancer [**10**,**11**].

A promising clinical research in breast cancer is the use of immune checkpoint inhibitors that work by targeting molecules that serve as checks in the regulation of immune responses and block inhibitory molecules or activate stimulatory molecules. There are some phase I/ II studies that enrolled patients with breast cancer in different stages of the disease, for treatments with indoximod, an IDO inhibitor (IDO is expressed by a number of tumor types and correlates with poor prognosis), an anti-OX40 antibody (OX40 is a costimulatory molecule expressed after T cell activation that enhances T cell survival and anti-cancer effector function) and an anti-PD-L1 checkpoint inhibitor.

Monoclonal antibodies (mAbs) are generated in the lab and target specific antigens on tumors. Many antibodies are currently used in cancer treatment. New antibodies are tested in breast cancer: glembatumumab vedotin - an antibody-drug conjugate used in patients with advanced triple-negative breast cancer with cells that produce a protein called glycoprotein NMB, margetuximab, an anti-HER2 antibody used in patients with relapsed or refractory advanced breast cancer with cells that express HER2 at the 2+ level and lack HER2 gene amplification by FISH. Cancer vaccines elicit an immune response against tumor-specific or tumor-associated antigens. Several trials of vaccines, given alone or with other therapies, are currently enrolling breast cancer patients: NeuVax is under investigation to prevent breast cancer recurrence among patients with HER2 1+ and 2+ following surgery. GVAX, a therapeutic vaccine made from breast cancer cell lines irradiated and engineered to express the immune molecule GM-CSF, is being tested in a phase II trial [**12**].

According to the method called adoptive T cell transfer (**Fig. 6**), T cells are removed from the patient, genetically modified, or treated with chemicals that enhance their activity, and re-introduced into the patient. There are several trials using adoptive T cell transfer techniques that are currently enrolling patients with breast cancer: an anti-HER2 bi-armed activated T cells after second line chemotherapy in women with HER2-negative metastatic breast cancer, a chimeric antigen receptor (CAR) T cells targeting cMet (abnormally activated in cancer and correlated with poor prognosis) and last, but not least, a T cell genetically engineered to target carcinoembryonic antigen (CEA) (prominently expressed in breast cancer) [**13**].

**Fig. 6:**
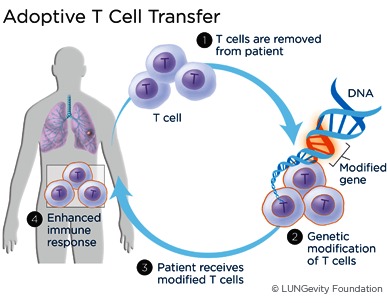
Adoptive T cell transfer

Cervical cancer is another cancer type for which new immune-based cancer treatments are currently under development [**14**].

The most significant cause of cervical cancer, as well as other ano-genital cancers, is the infection with the human papillomavirus (HPV). HPV is thought to cause more than 90% of the cases of cervical cancer, 90% of the anal cancers, 75% of the vaginal cancers, 70% of the vulvar cancers, and 60% of the penile cancers. HPV is also a significant cause of head and neck cancers.

Cervical cancer is the third most frequently diagnosed cancer among women worldwide and the second most frequently diagnosed cancer among women in Romania (**Fig. 7**). For the early stage disease, the 5-year survival rate is 91% and for patients with regional and distant disease is 57% and 16%, respectively.

**Fig. 7:**
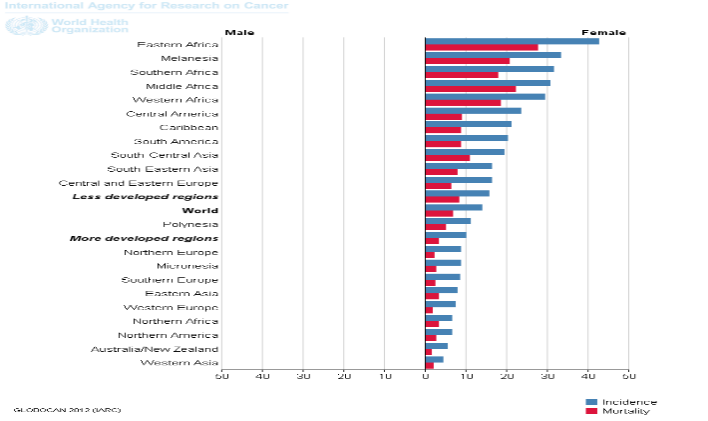
Cervical cancer: incidence and mortality rates among women

Cervical cancer can be diagnosed in the early stages of the disease with the screening Pap tests. Mortality rates have been decreasing thanks to the widespread use of the Pap test as a screening tool. There are approved vaccines such as Gardasil, Gardasil-9, and Cervarix that prevent HPV infection and lead to the furthermore decrease of cervical cancer incidence and mortality in countries that have implemented vaccination as a public policy. For people already infected with HPV and having a diagnosis of cervical or ano-genital cancer, these vaccines are of no use.

Immunotherapy agents designed to treat these types of cancers are badly needed [**15**]. Several checkpoint inhibitors that target multiple different checkpoints are currently under development. Ipilimumab, an anti-CTLA-4 antibody, is being tested in two clinical trials that enroll patients with cervical cancer; an OX40 immune modulator is being tested in combination with tremelimumab, an anti-CTLA-4 antibody; Urelumab-an anti-4-1BB/ CD137 antibody and nivolumab, an anti-PD-1 antibody; Lirilumab - an anti-KIR antibody, is being tested in combination with nivolumab; an anti-GITR antibody is also tested.

In adoptive T cell transfer, T cells are removed from a patient, modified to enhance their activity, and re-introduced into the patient with the goal of improving the T cell immune system’s anti-cancer response. A phase II study of white blood cells taken from the patient’s own tumor enrolls patients with HPV-related cancers, including cervical cancer [**16**].

Monoclonal antibodies (mAbs) are molecules that target specific antigens on tumors. Bevacizumab, which targets the vascular endothelial growth factor (VEGF), is approved for the treatment of recurrent or late-stage cervical cancer. An anti-cancer stem cell antibody that binds to the Notch1 receptor is tested in a phase I trial [**17**].

Cancer vaccines are designed to elicit a T cell immune response against tumor-specific or tumor-associated antigens. There are several studies that enroll patients with breast cancer into different stages to receive VGX-3100, a vaccine that targets HPV types 16 and 18; INO-9012, a DNA construct that induces human interleukin 12 (IL-12); ADXS11-001, a vaccine against the E7 protein made by HPV; MEDI4736, a PD-L1 inhibitor; a pNGVL4a/ E7 (Detox)/ HSP70 DNA vaccine in patients with HPV16+ cervical intraepithelial neoplasia, a combination with imiquimod (an innate immune activator) [**18**].

Brain cancer is another cancer type that desperately needs new treatments with high response rates. New immune-based treatments are currently under development in brain cancer tumors treatment [**19**]. There are several types of brain cancer, classified according to the type of cell from which they originate. Gliomas, which originate in glial cells that support and protect neurons, account for more than 70% of the brain cancers. Astrocytomas have origins in astrocytes. Meningiomas are tumors that begin in the meninges of the brain and spinal cord. The mortality rate for brain cancer has remained increased for more than 30 years, although significant advances have been made in understanding the biology of brain cancers.

Glioblastoma (GBM) is the most aggressive form of brain cancer. GBM patients typically have short life expectancies. Median progression free survival is of 6.9 months, and the median overall survival is of 14.6 months. In 2005, temozolomide was approved to treat GBM. In 2009, bevacizumab was granted accelerated approval for the treatment of GBM patients but there was no evidence of improvement in the overall survival.

Current immunotherapies that are in study in brain cancer fall into six broad categories: cancer vaccines, checkpoint inhibitors, oncolytic virus therapy, adoptive cell therapy, and monoclonal antibodies [**20**].

Checkpoint Inhibitors work by targeting molecules that serve as balances on immune responses. By blocking these inhibitory molecules, these treatments are designed to enhance pre-existing anti-cancer immune responses. There are at least 3 clinical studies that use checkpoint inhibitors such as: phase III trial testing Nivolumab and Iipilimumab—anti-PD-1 and anti-CTLA-4 antibodies, respectively—in patients with recurrent glioblastoma; a phase II trial testing Durvalumab, an anti-PD-L1 antibody, in patients with glioblastoma and a phase II trial testing Pembrolizumab, an anti-PD-1 antibody, with or without Bevacizumab, in patients with recurrent glioblastoma multiform.

Adoptive Cell Therapy consists in removing immune cells from a patient and genetically modifying them to enhance their immune activity, and then re-introduce them into the patient. There are only phase I/ II studies using an anti-EGFRvIII chimeric antigen receptor (CAR) T cells.

Cancer Vaccines in brain tumors are under development and use in clinical studies. Rindopepimut, a therapeutic vaccine targeting a mutant peptide called EGFRvIII, which is expressed in approximately one-third of the glioblastoma tumors, has a survival benefit among recurrent patients. An international phase III trial of rindopepimut in newly diagnosed glioblastoma completed its enrollment in December 2014. There are multiple phase I, II and III trials that use different types of vaccines in Brain tumors.

Oncolytic virus therapy uses a modified virus that can cause the self-destruction of tumor cells by releasing more antigens, generating a greater immune response against the cancer. There are only phase I studies regarding this therapy.

Monoclonal antibodies, generated in the lab, are molecules that target specific antigens on tumors. There are phase I/ II trials using anti-endoglin antibody or antibody-drug conjugate (ADC) that targets EGFR/ EGFRvIII [**21**].

Colorectal cancer is the third most common type of cancer among both men and women and is the second most deadly (**Fig. 8**). Over 95% of the colorectal cancers are adenocarcinomas. Overall death and incidence rates among men and women have been declining in the past years, thanks to the screening tests used in developed countries. In the early stage of the disease, the 5-year survival rate is 90% and, in the locally advanced stage, the 5-year survival rate is 71%, declining at 13% in the metastatic disease stage. 

**Fig. 8:**
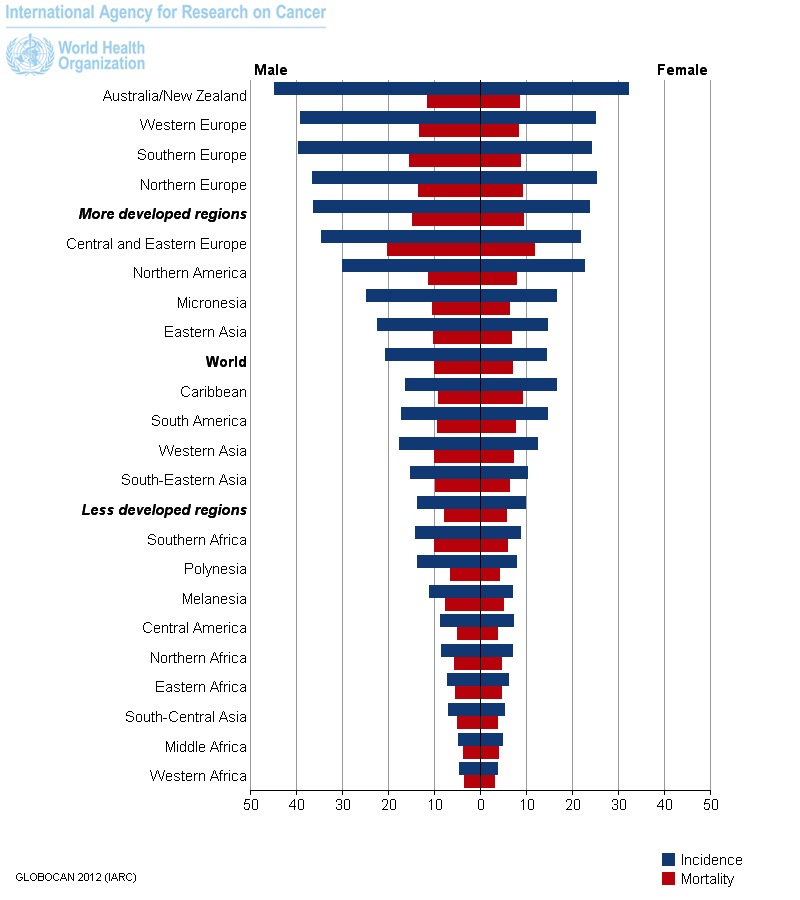
Colorectal cancer incidence and mortality rates among men and women

Personal or family history of colorectal cancer (or polyps), chronic inflammatory bowel disease, or genetic disease such as Lynch syndrome or familial adenomatous polyposis have been associated with an increased risk. 

The key treatment for colorectal cancer is surgery. Neoadjuvant or adjuvant chemotherapy, sometimes in combination with radiation therapy, is administered before or after surgery according to the disease guidelines. Standard first-line chemotherapy for metastatic colon cancer consists in several regimens containing fluoropyrimidine derivate, the most commonly used regimen being one of the following regimens: FOLFOX (5-FU, leucovorin, and oxaliplatin) or FOLFIRI (5-FU, leucovorin, and irinotecan). Adding bevacizumab may also help improve the survival. Cetuximab and panitumumab have also been shown to improve survival in patients with wild type RAS tumors. Aflibercept was approved for the second line treatment with chemotherapy. In September 2012, regorafenib was approved as a drug that targets multiple tyrosine kinases, for patients who progressed on prior therapy.

Current immunotherapies for colorectal cancer categories are the following: monoclonal antibodies, checkpoint inhibitors, cancer vaccines, adoptive cell therapy, oncolytic virus therapy, cytokines, and adjuvant immunotherapies [**22**].

Checkpoint inhibitors tested in clinical trials (phase I/ II), that enroll colorectal cancer patients are the following: pembrolizumab, a PD-1 antibody for patients with microsatellite unstable (MSI) tumors, including colorectal cancer; MEDI4736, an anti-PD-L1 checkpoint inhibitor; MEDI6469, an anti-OX40 agonist antibody, alone or with tremelimumab, an anti-CTLA-4 antibody, and/ or MEDI4736, nivolumab an anti-PD-1 antibody, +/ - ipilimumab, an anti-CTLA-4 antibody; urelumab, an anti-4-1BB/ CD137 antibody, tested in combination with nivolumab (anti-PD-1); MEDI6383, an anti-OX40 antibody; MEDI0680 (AMP-514), an anti-PD-1 antibody; and MEDI4736, urelumab, an anti-4-1BB/ CD137 antibody; varlilumab (CDX-1127), an anti-CD27 antibody; MOXR0916, an anti-OX40 antibody; lirilumab, an anti-KIR antibody; TRX518, an anti-GITR antibody [**23**].

Cancer vaccines in colorectal cancer elicit an immune response against tumor-specific or tumor-associated antigens. Tumor antigens that have been targeted in colorectal cancer include carcinoembryonic antigen (CEA), MUC1, guanylyl cyclase C, and NY-ESO-1. There are several clinical studies that enroll patients with colorectal cancer to receive cancer vaccines such as: Imprime PGG in combination with cetuximab in subjects with recurrent or progressive KRAS wild type colorectal cancer; a vaccine that targets the NY-ESO-1 protein in patients with advanced cancer whose cancers express NY-ESO-1; DCVax; FANG; AVX701, which targets the CEA antigen that has been found to be associated with colorectal cancers; a vaccine targeting brachyury, which helps drive cancer metastasis vaccine therapy with or without sirolimus in treating patients with NY-ESO-1 expressing solid tumors; a vaccine that targets the HER2 antigen in patients with metastatic cancer, including colorectal cancer [**24**].

Oncolytic virus therapy uses a modified virus that causes tumor cells self-destruction and generates a greater immune response against the colorectal cancer. Reolysin is a virus that is able to replicate specifically in cancer cells bearing an activated RAS pathway, in patients with metastatic colorectal cancer.

Cytokines are messenger molecules that help control the growth and activity of immune system cells. AM0010, a recombinant human interleukin 10 (IL-10), interleukin 15 (IL-15) and interleukin 12 (IL-12) are recently used in colorectal cancer studies.

Monoclonal antibodies tested in the new clinical trials in colorectal cancers are RO5520985, a bispecific anti-ANG-2/ anti-VEGF-A antibody; Sym004, an antibody targeting the cancer antigen EGFR; IMMU-132, an antibody-drug conjugate targeting Τrop-2; IMMU-130, an antibody-drug conjugate targeting CEACAM5; ensituximab (NPC-1C), an antibody against a MUC5AC-related antigen; bavituximab, an antibody that targets an immune-suppressing molecule in tumors; MORAb-066, targeting tissue factor (TF), an antigen overexpressed in tumor cells and tumor endothelial cells, in patients with TF-expressing cancers; anti-MIF antibody, which targets macrophage migration inhibitory factor; MGD007, a dual-affinity re-targeting (DART) protein designed to target the glycoprotein A33 antigen, which is found on 95% of the colorectal cancers. All studies are in phase I/ II of trial.

Adoptive Cell Therapy in colorectal cancer includes: tumor-infiltrating lymphocytes (TILs) in metastatic digestive tract cancers; T cells engineered to target VEGFR in patients with metastatic cancer; T cells engineered to target MAGE-A3 in patients with metastatic cancer that expresses MAGE-A3; T cells targeting EGFR in patients with advanced cancer, including colorectal cancer; natural killer (NK) cells, important innate immune cells, in patients with advanced cancer [**25**].

There are two main types of esophageal cancer: squamous cell carcinoma and adenocarcinoma. Risk factors for esophageal cancer include smoking tobacco and heavy alcohol use. These factors induce gene mutations that could be susceptible of immune recognition, therefore could be treated with immune modulators. Standard treatments in esophageal cancer do not obtain a good enough survival. Surgery is the most common treatment for esophageal cancer. Chemotherapy, radiation, and targeted therapies may also be used. For patients with HER2-positive esophageal tumors, the targeted therapy trastuzumab may be used in addition to chemotherapy. The 5-year relative survival rate for patients with esophageal cancer is 39% for patients with a localized disease; 21% for a regional disease; and 4% for a metastatic disease. 

Several approaches to immunotherapy for esophageal cancer have shown promising in early clinical trials: checkpoint inhibitors/ immune modulators, therapeutic vaccines, adoptive T cell transfer, monoclonal antibodies and cytokines [**26**,**27**].

Immune checkpoint inhibitors used in studies of esophageal cancer are the following: Nivolumab an anti-PD-1 antibody is being tested in a phase II trial for patients with advanced esophageal cancers; Pembrolizumab is also being tested; MPDL3280A, an anti-PD-L1 antibody is being tested; Urelumab and PF-05082566 an anti-4-1BB/ CD137 antibody is being tested; Mogamulizumab, an anti-CCR4 antibody, is being developed in a phase I trial for patients with advanced solid tumors.

Several trials of adoptive T cell transfer techniques are currently enrolling patients with esophageal cancer, including: a phase II trial taking enriched tumor-infiltrating immune cells and re-infusing them in patients with metastatic digestive tract cancers, including esophageal cancer, a phase II study of T cells genetically reengineered to target the NY-ESO-1 antigen in patients with NY-ESO-1-positive cancers; a phase I/ II trial of T cells genetically reengineered to target the anti-MAGE-A3-DP4 protein; a phase I/ II study of chimeric antigen receptor (CAR) T cells designed to target VEGFR2 [**28**,**29**].

Monoclonal antibodies (mAbs) molecules that target specific antigens on esophageal cancer tumors are currently under investigation and some appear to generate an immune response. Some new monoclonal antibodies are tested in clinical trials: MM-111, a bispecific antibody that binds to HER2 and HER3, in patients with esophageal and gastroesophageal junction cancers; IMMU-132, an antibody-drug conjugate targeting Τrop-2, in patients with esophageal and other cancers; ontuxizumab (MORAb-004), an antibody targeting endosialin/ TEM1; OMP-52M51, anti-Notch1 monoclonal antibody, in patients with solid tumors; ABT-700, an anti-C-met antibody, in patients with solid tumors; MM-151, an antibody designed to bind and inhibit signaling from EGFR, in patients with solid tumors; CEP-37250/ KHK2804, an antibody targeting glycolipids, in patients with advanced solid tumors.

Cancer vaccines are designed to elicit an immune response against tumor-specific or tumor-associated antigens and are tried in esophageal cancer. Several trials of vaccines, given alone or with other therapies, are currently enrolling patients: a vaccine that targets the NY-ESO-1 protein, ISCOMATRIX in patients with esophageal cancer; DCVax in patients with solid tumors, a vaccine (FANG) that blocks furin protein production, plus GM-CSF, for advanced cancer; NY-ESO-1 fusion protein vaccine in patients with advanced cancer, whose cancers express NY-ESO-1 [**30**].

Cytokines are messenger molecules that help control the growth and activity of immune system cells. The following are being tested: interleukin 15 (IL-15) in patients with advanced cancer, interleukin 12 (IL-12) in patients with esophageal cancer. 

The most common type of cancer of the head and neck is squamous cell carcinoma. Its incidence is increased in patients who smoke, drink alcohol, and have HPV infections, factors that determine gene alterations that can be translated into different proteins on the surface of cells that could activate the immune system, so studying immune treatments seems to be appropriate in these kinds of cancers [**31**].

Head and neck cancer is highly curable if detected early. More advanced head and neck cancers are generally treated with multi-modality surgery, including various combinations of surgery, radiation, and chemotherapy. 

Cancer vaccines encourage the immune system to attack cancer cells bearing tumor-specific or tumor-associated antigens. The following vaccines are being tested: AlloVax, a chaperone protein cancer vaccine; INO-3112 (VGX-3100 plus INO-9012, a DNA-based IL-12) in head and neck cancer caused by human papillomavirus (HPV) types 16 and 18 and ADXS-HPV [**32**].

The immune checkpoint inhibitors studied are the following: Nivolumab, Ipilimumab, and Urelumab, which is an anti-4-1BB/ CD137 antibody [**33**].

The monoclonal antibody studied in the head and neck cancer is MEHD7945A, an anti-HER3/ EGFR human monoclonal antibody. 

Adoptive T cell transfer techniques are also tested into different phase I/ II studies: tumor infiltrating lymphocytes (TILs) for human papillomavirus-associated cancers, including head and neck cancer and chimeric antigen receptor (CAR) T cell transfer [**34**].

The majority of kidney cancer cases are represented by renal cell carcinomas. In local kidney cancer, the 5-year survival rate is 92%. Metastatic kidney cancer has a 5-year-survival rate of 12%. The risk factors for kidney cancer include tobacco use, obesity, high blood pressure, chronic renal failure, exposure to certain industrial chemicals and radiation. Most of the renal cell carcinomas are a subtype called clear cell carcinoma. 

Surgery is the primary treatment for most kidney cancers. Kidney cancer is chemo and radio resistant. Targeted therapies and immune-based treatments are important components of treatment for advanced kidney cancer. Several targeted therapies have been approved in advanced kidney cancer, such as bevacizumab and sunitinib, temsirolimus and everolimus, which block a protein called mTOR, axitinib, sorafenib, pazopanib. Targeted therapies are often the first line of treatment for advanced kidney cancer. 

Patients with metastatic kidney cancer occasionally experience spontaneous regressions of metastatic disease after the surgical removal of the primary tumor and this was the first clue that determined medical doctors and researchers to think of immunotherapy in kidney cancer [**35**].

Cytokines have been used for more than a decade to treat kidney cancer: interleukin-2 (IL-2) and interferon-alpha with response rates of 10%-20%. IL-2 can have serious side effects and is used at present for cancers that do not respond to targeted therapies.

Several newer immunotherapies have become important in the treatment of kidney cancer: checkpoint inhibitors and immune modulators, cancer vaccines, adoptive cell therapy, monoclonal antibodies, and cytokines [**36**].

The checkpoint inhibitors that are studied in kidney cancer are the following: pembrolizumab, used for patients with advanced urothelial cancer, including the renal pelvis; nivolumab with ipilimumab versus sunitinib (Sutent), for patients with previously untreated advanced or metastatic renal cell carcinoma; MPDL3280A, a PD-L1 antibody, as monotherapy or in combination with bevacizumab versus sunitinib in patients with renal cell carcinoma; MPDL3280A, tremelimumab (anti-CTLA-4) and MEDI4736 (anti-PD-L1); varlilumab (CDX-1127), an anti-CD27 antibody; MGA217, an antibody that targets B7-H3; SGN-CD70A, an antibody that targets CD70; lirilumab, a KIR antibody, in combination with nivolumab; BMS-986016, a LAG-3 antibody, with or without nivolumab; urelumab, a 4-1BB/ CD137 antibody; TRX518, a GITR antibody. 

Some cancer vaccines used in kidney cancer include the following: dendritic cell immunotherapy AGS-003; DCVax, a vaccine therapy with or without sirolimus in treating patients with NY-ESO-1 expressing solid tumors [**37**].

The monoclonal antibodies currently being tested in clinical trials are the following: sonepcizumab, a monoclonal antibody that binds to and inhibits the function of sphingosine-1-phosphate (S1P), which promotes blood-vessel formation (angiogenesis); TRC105, an antibody targeting endoglin, overexpressed on endothelial cells and which is essential for angiogenesis; LY2875358, an antibody targeting mesenchymal-epithelial transition factor (MET), involved in tumor cell proliferation and resistance; IMMU-132, an antibody-drug conjugate targeting Τrop-2.

Cytokines that are used in clinical practice (IFN, IL2) and in clinical trials are the following: AM0010, a recombinant human interleukin 10 (IL-10), interleukin 15 (IL-15), interleukin 12 (IL-12).

The following immune cells can be removed from a patient, can be genetically modified and re-introduced into the patient and used in kidney cancer in clinical studies: CD8+ cells anti-VEGFR2 gene engineered; natural killer (NK) cells, important innate immune cells, in patients with kidney cancer.

In conclusion, from the preventive vaccine for cervical cancer to the first therapy ever proven to extend the lives of patients with metastatic melanoma, immunology has already led to major treatment breakthroughs for a number of cancers.

Immunotherapy is a major class of drugs for cancer therapy, knowing today that tumors mediate immunosuppression through cellular mechanisms, which protect tumors from immune system recognition or rejection. Probably, in time, immunotherapy alone or together with target therapy will lead to personalized medicine. That will change completely the overall survival and progression free survival for many treatments. 
